# The Cholinergic Anti-Inflammatory Response and the Role of Macrophages in HIV-Induced Inflammation

**DOI:** 10.3390/ijms19051473

**Published:** 2018-05-16

**Authors:** Manuel Delgado-Vélez, José A. Lasalde-Dominicci

**Affiliations:** 1 Molecular Sciences Research Center, University of Puerto Rico, San Juan 00926, Puerto Rico; manuel.delgadovelez@upr.edu; 2 Department of Biology, University of Puerto Rico, Río Piedras Campus, San Juan 00931, Puerto Rico; 3 Department of Chemistry, University of Puerto Rico, Río Piedras Campus, San Juan 00931, Puerto Rico

**Keywords:** inflammation, macrophage, alpha7 acetylcholine receptor, cholinergic anti-inflammatory response, HIV, inflammatory response, cholinergic receptors

## Abstract

Macrophages are phagocytic immune cells that protect the body from foreign invaders and actively support the immune response by releasing anti- and proinflammatory cytokines. A seminal finding revolutionized the way macrophages are seen. The expression of the neuronal alpha7 nicotinic acetylcholine receptor (α7-nAChR) in macrophages led to the establishment of the cholinergic anti-inflammatory response (CAR) in which the activation of this receptor inactivates macrophage production of proinflammatory cytokines. This novel neuroimmune response soon began to emerge as a potential target to counteract inflammation during illness and infection states. Human immunodeficiency virus (HIV)-infected individuals suffer from chronic inflammation that persists even under antiretroviral therapy. Despite the CAR’s importance, few studies involving macrophages have been performed in the HIV field. Evidence demonstrates that monocyte-derived macrophages (MDMs) recovered from HIV-infected individuals are upregulated for α7-nAChR. Moreover, in vitro studies demonstrate that addition of an HIV viral constituent, gp120_IIIB_, to uninfected MDMs also upregulates the α7-nAChR. Importantly, contrary to what was expected, activation of upregulated α7-nAChRs in macrophages does not reduce inflammation, suggesting a CAR disruption. Although it is reasonable to consider this receptor as a pharmacological target, additional studies are necessary since its activity seems to differ from that observed in neurons.

## 1. Introduction

In the 19th century, insight into the role of macrophages in human immunology started to emerge [1]. Initially, they were identified as phagocytic cells. Later, technological advances coupled with the advent of molecular biology, proteomics, and genomic approaches allowed a better understanding of the complexities of these unique “warriors” that collaborate with the immune system to protect the host. Beyond being entirely phagocytic cells that remove dead cells and cellular or exogenous debris, macrophages secrete pro- and anti-inflammatory mediators that enhance the immune response and ultimately participate in the destruction and removal of exogenous invading agents from the host’s system. However, when this does not happen, the host becomes ill.

Inflammation is a formidable response that protects the body from exogenous entities such as pathogens. Inflammation involves the attraction of leukocytes and extravasation of plasmatic proteins in the zones of infection, as well as the activation of these leukocytes and proteins to eliminate the infectious agent. Short-term and controlled inflammation processes are a part of an optimal immune response that allows the body to fight and eliminate potentially pernicious agents effectively. However, uncontrolled inflammation and chronic inflammation are life-threatening and are characteristic of chronic infectious diseases such as human immunodeficiency virus (HIV) infection. Indeed, people infected with HIV suffer from chronic inflammatory processes that promote the appearance of non-acquired immunodeficiency syndrome (AIDS)-related complications [2,3]. Macrophages play a crucial role in HIV-induced inflammation and infection. Upon HIV infection, macrophages release cytokine cascades that promote viral replication, which in turn promotes accumulation of virotoxins (trans-activator of transcription (Tat), negative regulatory factor (Nef), viral protein R (Vpr), and envelope glycoprotein GP120 (gp120)) in the blood that further potentiate its viral latency and persistence. Over the past years, understanding of the biology of macrophages has changed dramatically, mainly because of the discovery of their participation in an innate neuroimmune response to control inflammation in mammals.

In the early 2000s, Kevin Tracey’s group [4] made a seminal discovery that changed the way the scientific community looks at the macrophage. They demonstrated that the macrophage is a key player in the cholinergic anti-inflammatory pathway (CAP). An immediate outcome of this important finding was that these cells began to be recognized as a crucial player that mediates anti-inflammatory processes as part of the neuroimmune connection called CAP. For this pathway, the central neural tract is the vagus nerve that, upon stimulation, releases acetylcholine (ACh) to further interact with a cholinergic receptor expressed by macrophages—the alpha7 nicotinic acetylcholine receptor (α7-nAChR) [4,5]. On the basis of this critical new role identified in macrophages, it was possible to interrogate a number of inflammatory processes and diseases, thus facilitating the development of new therapeutic strategies including development of new medical devices and novel therapeutic strategies to activate the CAP [6,7,8] in mammals. 

Reconciling the information available about studies aimed at understanding the status of CAP in HIV is of utmost importance because the chronic inflammation suffered by these patients continues to be an unresolved problem that urgently needs a solution to avoid the appearance of non-AIDS-related complications (i.e., osteoporosis, cardiovascular disease, neurocognitive deterioration, renal dysfunction, thromboembolic disease, insulin resistance and type II diabetes, cancer, multiple end-organ disease, and frailty [3,9,10,11,12]) in these individuals. The purpose of this review is to present the latest knowledge about the involvement of macrophages in HIV-induced inflammation, together with emerging work examining the cholinergic anti-inflammatory response (CAR) of macrophages in the context of HIV infection. Also, possible therapeutic pharmacological approaches are presented to target the CAR in HIV-related settings. We conclude that more work needs to be done focused on identifying and characterizing the α7-nAChR expressed by macrophages to gain knowledge about its pharmacological properties. It is well known that this receptor interacts with several proteins from the intracellular and transmembrane side, which must undoubtedly influence its activity. The information generated from these studies can result in the identification of a new therapeutic target to alleviate inflammatory problems for HIV-infected individuals. 

## 2. Monocytes and Macrophages: Their Origin and Role in Immune Response

Monocytes are the largest blood cells that belong to the mononuclear phagocyte system. This unique system comprises monocytes, macrophages, and dendritic cells, as well as their committed bone marrow progenitors [13,14]. They contain granules (primary lysosomes) and have a lobular-shaped nucleus. Also, monocytes phagocytose and have bactericidal activity. In circulation, human monocytes have a diameter varying from about 10 to 18 µm; the surface possesses microvilli and an undulating membrane. The heterogeneity in size may be explained by differences in maturation state and/or activation state. Monocytes originate in the bone marrow from pluripotent stem cells and mobilize entering the circulation. In humans, the monocyte pool comprises three subsets (classical, intermediate, and nonclassical) that circulate in dynamic equilibrium and can be differentiated based on CD14 and CD16 expression [15,16]. Thus, CD14^+^ CD16^−^ (classical) monocytes make up ∼85% of the circulating monocyte pool, whereas the other ∼15% consists of CD14^+^ CD16^+^ (intermediate) and CD14^low^ CD16^+^ (nonclassical) monocytes [17,18,19]. Under normal conditions, monocytes make up 3% to 8% of the circulating cell population, and their numbers increase in response to infection. The life span of a circulating monocyte is fairly brief, and most undergo apoptosis after about 24 h [20]. Recently, using a human in vivo deuterium labeling approach, a study that focused on determining the kinetics underlying the generation, differentiation, and disappearance of monocytes from these three subsets was elegantly performed. The authors demonstrated that classical monocytes emerge first from marrow, after a postmitotic interval of 1.6 days, and circulate for a day. Subsequent labeling of intermediate and nonclassical monocytes is consistent with a model of sequential transition. Specifically, intermediate and nonclassical monocytes have longer circulating lifespans (~4 and ~7 days, respectively) [17]. Furthermore, in the same study, using a human experimental endotoxemic model, the authors observed a transient but profound monocytopenia, and restoration of circulating monocytes was achieved by the early release of classical monocytes from bone marrow [17]. Some monocytes do, however, migrate into tissues or to the sites of damage or infection where they subsequently differentiate into macrophages, such as the Kupffer cells of the liver, microglia in the central nervous system (CNS), dermal macrophages in the skin, and splenic marginal zone and metallophilic macrophages. Importantly, it is now recognized that the majority of tissue macrophage populations are seeded before birth [17,21,22,23,24,25] and are maintained via self-proliferation throughout adulthood with minimal monocyte input. Of note, macrophages originate not only from the bone marrow but also from the fetal yolk sac and fetal liver. Accordingly, microglia and tissue-resident macrophages such as nonparenchymal CNS macrophages are derived from yolk sac, while bloodborne monocytes emerge from fetal liver. These different origins dictate diverse functions. 

The macrophage system was first introduced by the Russian scientist Élie Metchnikoff in the late 19th century, who described its phagocytic cell activity [1] and won the Nobel Prize in 1908 for this and other discoveries [26]. The professional role of macrophages is to phagocytose foreign invaders such as microbes and extraneous particulate matter. Macrophages are resident phagocytic cells in lymphoid and nonlymphoid tissues and are involved in steady-state tissue homeostasis [27,28,29] via the clearance of apoptotic cells, the production of growth factors [30], the recycling of nutrients by digesting waste products from tissues [31], and the secretion of pro- and anti-inflammatory mediators [32]. Macrophages can express a broad range of pathogen recognition receptors that make them efficient at detecting danger, triggering an inflammatory response through production of inflammatory and anti-inflammatory cytokines (interleukins, chemokines) that modulate/potentiate the immune response through activation of other immune cells that ultimately generate a specific immune response against a specific foreign invader. Also, they can be stimulated by cytokines secreted by T helper cells, with interferon gamma (IFN-γ) being one of the most potent macrophage activators.

The chemical, immunological, and cellular environment where the macrophages reside influences their inflammatory profiles and dictates their phenotypes. Monocyte-derived macrophages can be specialized in response to cell stimulations from the organ where they reside, microbial products from pathogens (i.e., lipopolysaccharides (LPS)), and activated lymphocytes. These cues and factors are responsible for modifying macrophage’s metabolism, allowing the polarization of them into two distinctive phenotypes, M1 and M2, which exhibit different inflammatory profiles [33]. The M1 macrophages arise thanks to the stimulating activity of molecules such as LPS, interferon gamma, granulocyte-macrophage colony-stimulating factor, and tumor necrosis factor alpha (TNF-α). Once polarized (activated), M1 macrophages secrete proinflammatory cytokines (interleukin (IL)-1β, TNF, and IL-6) and toxic effector molecules such as reactive oxygen species and nitric oxide. Conversely, M2 macrophages emerge from IL-4 or IL-13 cytokine stimulation and secrete significant levels of the anti-inflammatory cytokine IL-10 and low levels of IL-12. Notably, M2 macrophages encompass a functionally diverse group of cells with different activation states [34]. Accordingly, the M2a macrophages emerge in response to IL-4 and IL-13 (alternative inflammation) stimulation, M2b macrophages emerge from Fc-gamma receptors/toll-like receptor (FcγR/TLR) activation triggered by immune complexes and LPS, M2c macrophages result from IL-10, transforming growth factor beta (TGF-β), and glucocorticoids which promote deactivation, and, finally, M2d activation is a response to IL-6 and adenosines [35,36].

Once a pathogen gains access to the body, either by an injury (wound) or through some of the entry portals of the body (mucous membranes, skin, respiratory and gastrointestinal tracts), among the first cells with which these invaders interact are macrophages. Thus, the immune response starts with pathogen recognition through the macrophage’s Toll-like receptors, mannose receptors, or scavenger receptors that have broad ligand specificity for lipoproteins, proteins, oligonucleotides, polysaccharides, lectins, and other molecules expressed by pathogens [31]. In fact, the mannose receptor is a cell-bound C-type lectin that binds certain sugar molecules found in the HIV. In mammals, a Toll-family protein, called Toll-like receptor 4 (TLR-4), signals the presence of LPS by associating with CD14, the macrophage’s receptor for LPS [37]. In addition to these functions, macrophages express the major histocompatibility complex class II (MHC II), which presents antigens to lymphocytes. Accordingly, once a macrophage engulfs a pathogen, its antigens are processed, transported, and presented to T helper cells [31] through the MHC II. Then, T cells release cytokines that, in turn, activate B cells, resulting in antibody production. These antibodies recognize specific antigens from the pathogen that triggered the immune response. Finally, the antigen–antibody complexes are avidly recognized by macrophages to be phagocytosed and ultimately destroyed [31]. For a comprehensive review detailing the phagocytic diversity and versatility of macrophages, see Lim et al., 2017 [38]. In summary, monocytes and macrophages, which are part of the body’s cellular surveillance system that protects us from exogenous agents, enhance the immune response to eradicate such agents before they are established in the host.

## 3. Macrophages, Inflammation, and HIV Infection

Once a foreign invader gains access to the body, the immune system mounts a unique response to destroy it. This response depends on the nature of the pathogen involved: fungi, bacteria, yeast, parasite, protozoa, or virus. As mentioned, our discussion will be focused on the HIV, a virus responsible for the death of 1 million people in 2016 [39] and 1.8 million new infections per year globally [40]. In fact, since the discovery of HIV, a total of 35 million individuals have died from the acquired immunodeficiency syndrome (AIDS) caused by it [39].

After HIV has entered the body, a molecular recognition dynamic is initiated between the viral gp120 and CD4-expressing cells. Macrophages express not only the CD4 receptor but also C-X-C chemokine receptor type 4 (CXCR4) and C-C chemokine receptor type 5 (CCR5) coreceptors necessary for efficient HIV entry and productive infection [41,42,43,44,45,46]. Early immune and virological events during HIV infection are associated with disease progression [47,48]. Peak viremia is accompanied by immune system activation and the lamina propria’s CD4^+^ T cell destruction in the gastrointestinal tract, which is the first vital system colonized by HIV during acute infection. It is now clear that the mucosal immune system and the intestinal immune system are pivotal to the pathogenesis of AIDS because most of the critical events of transmission, viral amplification, and CD4^+^ T cell depletion occur in the gastrointestinal tract [49,50]. In time, although viral load subsequently declines, immune activation continues, HIV replication continues, and CD4^+^ T cells are progressively destroyed and lost [51,52]. Immune activation during HIV infection involves activation and proliferation of multiple cells of immune system, including T cells, B cells, natural killer cells, and macrophages [49,50]. Along with the gastrointestinal changes and abrupt cellular activation, gut dysbiosis is characteristic of chronic HIV infection [53]. Accordingly, translocation of microbial products (i.e., LPS) and, potentially, microbes from the intestinal lumen into systemic circulation has been linked to immune activation, inflammation, severity of HIV-/simian immunodeficiency virus (SIV)-associated chronic immune activation, and HIV-1 disease progression [53,54,55,56,57]. Macrophages play an important role in amplifying the immune response and inflammation during HIV infection in the presence of microbial products or microbes from leaking gut. The latter is associated with persistent macrophage activation independent of viral replication or T cell activation in pediatric patients [58]. On the other hand, quantification of serum biomarkers of microbial translocation (LPS) and macrophage activation markers (soluble CD14 (sCD14)) predict subclinical atherosclerosis progression in HIV-infected adults [59], a common comorbidity suffered by HIV-infected individuals.

Specifically, during acute infection, HIV-infected individuals experience an intense proinflammatory cytokine cascade [60] that predicts disease progression [61,62]. This excessive secretion of cytokines, upon infection, is a turning point that marks the initiation of an irreversible systemic inflammatory environment. The release of cytokines during acute infection is characterized by rapid and transient elevations in interferon alpha (IFN-α) and interleukin-15 (IL-15) levels followed by a significant increase in inducible protein 10 (IP-10) levels; rapid and more-sustained increases in tumor necrosis factor alpha (TNF-α) and monocyte chemotactic protein 1 (MCP-1) levels; more slowly initiated elevations in levels of additional proinflammatory factors including IL-6, IL-8, IL-18, and IFN-γ; and a late-peaking increase in levels of the immunoregulatory cytokine IL-10 [60]. Eventually, as the infection progresses, the cellular and chemical environment changes and becomes chronic. As a consequence, HIV-infected individuals suffer from chronic and persistent inflammation [3,60,61,63,64,65,66,67] that promotes the appearance of non-AIDS-related complications such as osteoporosis, cardiovascular disease, neurocognitive deterioration, renal dysfunction, thromboembolic disease, insulin resistance and type II diabetes, cancer, multiple end-organ disease, and frailty [9,10,11]. These comorbidities arise as a consequence of a highly inflammatory cellular environment that is linked to the macrophages’ secreted cytokines and chemokines during the immune response. Moreover, these non-AIDS-related complications occur despite antiretroviral therapy [68,69,70,71]. 

As mentioned, HIV-infected individuals develop several non-AIDS life-threatening complications derived from the infection itself that are considered comorbidity factors, although recently this has been referred to as a multimorbidity because of the simultaneity of multiple diseases occurring during HIV infection [72]. These comorbidity factors are associated with the response of macrophages to infection. This being so, HIV-infected individuals suffer from bone loss (osteoporosis) that is characterized by elevated levels of sCD14, a marker of macrophage activation. Interestingly, this phenomenon is more marked in males (higher sCD4 levels) than in females, and it is characterized by an inverse correlation between bone mineral content and bone mineral density measures [73]. In the case of HIV-induced cardiovascular diseases, particularly atherosclerosis, it has been suggested that HIV infection of T cells and macrophages results in the induction of oxidative and endoplasmic reticulum stress followed by the formation of the inflammasome and eventual autophagy dysregulation that facilitates atherogenesis [74]. Moreover, a recent study, focused on determining whether macrophage inflammation biomarkers are related to carotid atherosclerosis, suggested that two macrophage inflammation markers—sCD14 and sCD163—play essential roles in atherogenesis among HIV-infected males and females [75]. Of note, in females, other inflammatory markers derived from macrophages also support the inflammatory scenario induced by HIV infection. Accordingly, in addition to sCD14 and sCD163, the macrophage inflammatory marker galectin-3-binding protein is significantly associated with carotid artery disease in examined females [76]. As mentioned before, neurocognitive deterioration is also experienced by HIV-infected individuals and is associated with monocytes/macrophages. In support of the role of inflammation, it has been shown that sCD14 (a macrophage inflammatory marker) correlates with the CD4 nadir, and both are strong predictors of the severity of neurocognitive impairment [77,78,79,80]. Also, macrophages act as a persistent reservoir of HIV, even under combination antiretroviral therapy (ART) and highly active antiretroviral therapy (HAART), and thus remain a source of systemic and brain inflammation [81,82,83]. Another vital system affected by HIV infection is the renal system. The two most common renal-associated diseases are HIV-associated nephropathy and HIV-immune-complex kidney disease. There is evidence of the involvement of macrophages in this system during HIV infection. A study using tubular epithelial cells isolated from biopsies or urine of children with HIV-associated nephropathy showed that HIV-1 virions from infected macrophages could be detected in membrane-bound, intracellular vesicles within the tubular cells when the two cell types were cultured together [84]. Moreover, unexpectedly, the host renal system seems to intensify inflammation since secretion of TNF-α and IL-6 from renal tubular cells was shown to enhance viral replication in HIV-infected macrophages [85]. The endocrine system is also affected during HIV infection, and macrophages play a pivotal role in potentiating local and systemic inflammation. Aligned with this is the finding that high levels of the macrophage inflammatory marker sCD163 are associated with insulin resistance in HIV-infected individuals [86]. Finally, HIV-infected people have a 60-times-higher risk of developing non-Hodgkin’s lymphoma (a cancer of the lymphatic system) than do noninfected people, despite HAART administration [87]; this is promoted by tumor-associated macrophages which are active viral reservoirs for HIV [88]. These findings taken together suggest that HIV infection of macrophages is a critical step toward the development of chronic inflammation and the eventual advance of comorbidity/multimorbidity factors in HIV-infected individuals. 

## 4. Macrophages and the Cholinergic Anti-Inflammatory Response: A Focus on α7-nAChR

**Figure 1:**
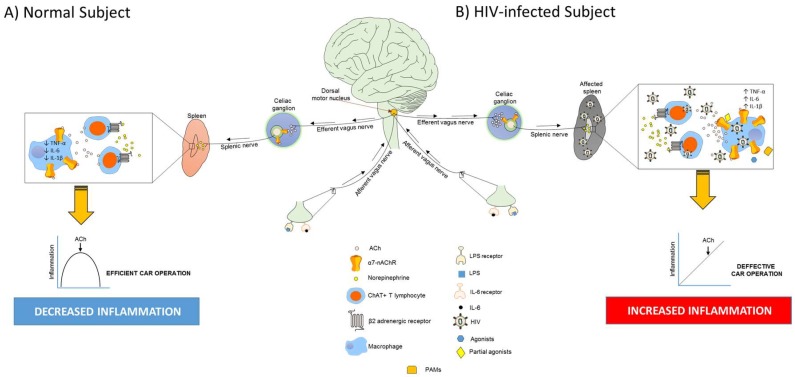
The cholinergic anti-inflammatory response (CAR) in normal and HIV-infected subjects. (**A**) During inflammation, the afferent vagus nerve recognizes microbial products (LPS) or cytokines (IL-6) that travel to the dorsal motor nucleus where they are interpreted and trigger an efferent response through the efferent branch of the vagus nerve. Accordingly, the efferent vagus nerve emerges from dorsal motor nucleus and innervates the celiac ganglion to release acetylcholine (ACh) that binds to α7-nAChRs in splenic neurons. Then, norepinephrine is release into the spleen via splenic nerve to activate β2-adrenergic receptors ChAT^+^ T lymphocytes which, in turn, secrete ACh that activates macrophage’s α7-nAChR, inhibiting the release of proinflammatory cytokines (i.e., TNF-α, IL-6, and IL-1β); (**B**) In HIV-infected subjects, the spleen and the CAR seem to be compromised and persistent inflammation is maintained. In fact, there is evidence demonstrating that addition of ACh does not decrease the production of proinflammatory cytokines in monocyte-derived macrophages despite elevated levels of α7-nAChR, suggesting a CAR disruption [107]. On the other hand, the α7-nAChR in macrophages could be exploited as a pharmacological option to treat inflammation by means of agonists, partial agonists, or positive allosteric modulators (PAMs).

Historically, macrophages were appreciated as phagocytic cells that protect the body from invasion by external agents, as well as cells that actively participate in the immune response via release of stimulatory cytokines and chemokines. However, this view was revolutionized by a series of reports demonstrating that macrophages express a neuronal nicotinic acetylcholine receptor known as α7-nAChR [4]. Agonist activation of the α7-nAChR expressed by macrophages is essential to reduce systemic inflammation through vagus nerve stimulation [5] that is part of a unique neuroimmune tract known as the cholinergic anti-inflammatory pathway (CAP) [4,5]. Compelling evidence demonstrates that the CAP regulates the innate immune system and can be activated artificially by applying electrical stimuli or pharmacologically via drug administration. Physiologically speaking, the CAP could be activated once the vagus nerve recognizes consumed activators such as those obtained nutritionally in the diet (lipids, peptides, and proteins). Importantly, inflammatory mediators (cytokines) secreted by immune cells during an immune response or exogenous bacterial constituents such as LPS can also activate the CAP. It is also known that microorganisms could activate the afferent vagus nerve directly at the level of the nodose ganglion during an infection because it expresses TLR4 [89]. To be more specific, a typical CAP activation occurs through afferent vagus nerve recognition of LPS, peripheral proinflammatory cytokines (i.e., IL-6, TNF-α, and IL-1β), immunoglobulins, or ATP through receptors expressed by the vagus nerve endings [90,91,92,93,94,95]. The signal travels from periphery to the brain where it is processed via a muscarinic/acetylcholine receptor-dependent mechanism [96,97,98] (Figure 1A). The integrated anti-inflammatory signal is conveyed through vagus nerve efferent fibers originating in the dorsal motor nucleus, connects to the splenic nerve in the celiac-superior mesenteric plexus (celiac ganglion), and conveys the anti-inflammatory signal to the spleen (Figure 1A). Here, the splenic nerve terminals release norepinephrine (NE), activating β2 adrenergic receptors in specialized T lymphocytes that express choline acetyltransferase (ChAT^+^ T cells) and synthesize acetylcholine (ACh) [99]. The NE-activated ChAT^+^ T cells travel to macrophages in the spleen and release ACh. Activation of the α7-nAChR in macrophages by ACh released by the ChAT^+^ T cells inhibits the production and release of proinflammatory cytokines (Figure 1A), whereas anti-inflammatory cytokine IL-10 levels remain unchanged [5].

Although α7-nAChR has been the most studied cholinergic receptor in macrophages, it is important to note that other nicotinic cholinergic receptors have been identified in mammalian primary cultures and monocyte/macrophage cell lines. For instance, the presence of α1, α4, α5, α6, α7, α9, α10, β1, β2, β3, and β4 subunits, at the mRNA level, has been demonstrated in the human monocytic cell line U937 [100]. Another group, using alveolar macrophages from mice, performed immunohistochemical studies that demonstrated the presence of α3, α4, α5, α7, α9, α10, β2, β3, and β4, while RT-PCR proved the existence of α3, α4, α5, α6, α9, α10, β2, β3, and β4 mRNAs [101]. Also, on macrophages obtained from a mouse blood cell line (RAW 264.7), mouse microglia primary cultures, and a mouse microglia cell line (N9), the presence of α7-nAChR was demonstrated through flow cytometry [102] and by RT-PCR and immunoblot [103]. Additionally, positive results were obtained for the presence of α3, α5, α9, α10, β1, and β2 in rat alveolar macrophages as determined by RT-PCR, immunohistochemistry, and immunoblot [104].

Interestingly, one study suggests that the professional function of macrophages—phagocytosis—requires α4β2-nAChR, but not α7-nAChR, as determined in mouse intestinal macrophages [105]. Another study found that tissue-resident macrophages in the mouse stomach express the β2 subunit but not α7-nAChR at the protein level, though those in the intestine express both receptor subunits [106]. Fortunately, all of these receptors exhibit different pharmacological properties that enable them to be studied separately, thus shedding light on their possible roles on macrophages. So far, the totality of roles and functions of each cholinergic receptor, as well as the possible effects they may have on the function of macrophages, remains poorly understood. For instance, it is still unknown whether the activation of α7-nAChRs expressed by macrophages established in different organs of the body has the same anti-inflammatory effect as those already identified in spleen’s macrophages that are associated with the CAP [4] (Figure 1A). Finally, from the clinical perspective, the role of α7-nAChRs expressed by macrophages during infection and inflammatory processes takes on special relevance regarding efforts to develop new therapeutic strategies to counteract disease states. HIV infection and its induced inflammation perfectly sum up the importance of macrophages, mainly because they are not only a target for HIV but also express α7-nAChRs. 

## 5. Macrophages, Inflammation, and the Cholinergic Anti-Inflammatory Response during HIV Infection

HIV infection is characterized not only by depletion of cells from the immune system but also by a chronic and persistent inflammation that promotes immunosenescence in these patients [67]. In HIV-infected individuals, inflammation occurs despite antiretroviral therapy, undetectable viral levels, and absence of symptoms. Inflammation starts soon after infection, and it is characterized by a dramatic cytokine cascade with plasma levels of some of the most rapidly induced cytokines peaking 7 days after the first detection of plasma viremia [60]. Eventually, it becomes a chronic inflammatory process that triggers a number of life-threatening non-AIDS-related comorbidities. A myriad of inflammatory markers has been identified in infected individuals that vary depending on the infection stage. These markers include cytokines, chemokines, and coagulation markers. Accordingly, during acute infection, marked levels of IP-10, IL-17, IL-12p40, IL-1α, eotaxin, granulocyte-macrophage colony-stimulating factor (GM-CSF), IL-12p70, IFN-γ, IL15, TGF-β1, and IL-18 have been identified; during chronic infection, elevated levels of neopterin, β2-microglobulin, D-dimer, C-reactive protein (CRP), TNF-α, TNF-RII, IL-6, LPS, sCD14, IL-10, and TGF-β1 are present [108]. T cell activation is also used as a biomarker predicting progression toward AIDS. Thus, CD8^+^ T cells expressing CD38 and human leukocyte antigen-antigen D related (HLA-DR) and the proportion of Ki-67 expressed by T cells have been used as bioindicators of HIV infection progression. Therefore, it is evident that the exaggerated release of inflammation mediators adds a further level of complexity to the already ongoing immune response and represents an even more significant challenge for the body of HIV-infected individuals. Among the cells that participate in this process are macrophages. These are important not only because HIV targets them, but also because they contribute to inflammation by releasing inflammatory mediators and participate in the CAR, an innate anti-inflammatory response mediated by a neuroimmune connection. Also, dendritic cells become infected by HIV and contribute to the overall inflammation. Specifically, during HIV infection, plasmacytoid dendritic cells release type-I interferons and proinflammatory cytokines to recruit more dendritic cells and CD4^+^ T cells to the site of HIV invasion (submucosa) [109]. Also, in addition to accumulating in the gut mucosa, plasmacytoid dendritic cells are associated with lymphoid tissue and contribute to immune activation by releasing proinflammatory cytokines [110]. Overall, the simultaneous presence of these proinflammatory mediators promotes a complex immunological response in which the net response is a state of constant, persistent, and chronic inflammation that facilitates viral establishment and replication and, together with immune cell destruction, promotes the appearance of immunodeficiencies and immunosenescence.

Unfortunately, available antiretroviral therapies neither eradicate HIV in its entirety nor eliminate chronic inflammation in infected individuals. Consequently, no functional cure exists at present. Hence, in the search for other ways to halt inflammation and prevent the non-AIDS conditions that result from it, the scientific community began to pay particular attention to a neuroimmune response that is part of the body’s innate immune response—the cholinergic anti-inflammatory response (CAR). Surprisingly, only recently has the relationship between α7-nAChR and the CAR begun to be studied in HIV-related scenarios, despite the fact that these patients suffer from chronic inflammatory problems (i.e., coagulopathies, vascular dysfunction, cardiovascular disease) that do not cease even under virological suppression [3,10,111].

The first clues linking HIV to nAChRs emerged from binding studies that showed the existence of homologous sequences between HIV-1 gp120, α-cobratoxin, and κ-bungarotoxin (selective nAChR antagonists) [112]. These observations were later strengthened by a study using the HIV_SF2_ isolates and several Swedish HIV-1 strains, which were precultivated from blood cells or cerebrospinal fluid (CSF) or were directly obtained from CSF cells of patients. Notably, this study found sequence similarities between a short segment of gpl20 of clinical HIV-1 strains and the snake venom neurotoxins [113]. Thus, the identified homology between the sequence 164–174 of HIV-1 gpl20 and the sequence 30–40 of snake venoms opened the possibility that HIV-1 indeed interacts (“binds”) with nAChR-expressing cells including muscle cells and neurons [114,115] and those immune cells targeted by HIV such as lymphocytes, monocytes, and macrophages, which also express the α7-nAChR essential for the CAP operation. Importantly, it is now well known that in monocytes and macrophages the activation of α7-nAChR inhibits the production of proinflammatory cytokines [4,116] as part of the CAR, something unidentified at the time these initial works were published.

While some studies have investigated nAChRs and HIV in the CNS [117,118,119,120,121,122,123,124,125,126], only two studies have explored the role of α7-nAChR at the peripheral level in a setting reminiscent of HIV infection [107,127]. Studies focused on the CNS have demonstrated that HIV and its viral constituents (i.e., Tat and gp120) promote an increase in calcium levels, leading to severe neurological alterations and even neuronal cell death [117,118,119,120,128]. On the other hand, studies focused on microglia (the resident macrophages of the CNS) have shown that nicotine and galantamine attenuate inflammation in vitro in an experimental setting that recapitulates HIV-associated dementia (HAD) [129]. Moreover, nicotine (an α7-nAChR agonist) increases the expression of HIV in microglia and alveolar macrophages [130,131], and, finally, α7-nAChR agonists attenuate gp120-induced mechanical allodynia and inflammation in a murine model [132]. 

As mentioned, two peripheral studies have explored the dynamic of nAChRs and HIV: in both cases, the α7-nAChR was the cholinergic receptor involved. The first study was focused on determining the mechanism by which HIV promotes lung diseases in HIV-infected individuals. Results identified two distinct pathways (α7-nAChR-GABAAR and epidermal growth factor receptor [EGFR]) for airway mucus formation and demonstrated for the first time that HIV-gp120 induces and regulates mucus formation in the airway epithelial cells through the CXCR4-α7-nAChR-GABAAR pathway [127]. Interestingly, the authors also found that although the gp120 immunoreactivity was widely scattered in simian immunodeficiency virus (SIV)-infected animals under antiretroviral treatment, the gp120 immunoreactivity was localized mainly in macrophage-like cells within the lungs. No further experiments were performed to determine the importance and role of these “macrophage-like cells” under the experimental design explored in this work. In the second study, the consequences of a CXCR4-tropic gp120_IIIB_ on α7-nAChR expression were determined using human blood monocyte-derived macrophages (MDMs). Results showed that the presence of pathophysiological amounts of gp120_IIIB_ induces the upregulation of α7-nAChR in a CXCR4-dependent manner [107]. Moreover, determination of α7-nAChR levels in MDMs recovered from HIV-infected individuals revealed that, in addition to monocytes and T cells, MDMs exhibit elevated levels of α7-nAChR protein, presumably triggered by soluble gp120_IIIB_. Importantly, this study tested, in vitro, the CAR in MDMs recovered from noninfected individuals, upregulated for α7-nAChR by gp120_IIIB_ and challenged with LPS. Accordingly, proinflammatory (TNF-α, IL-17, IL-6, MCP-1, regulated on activation, normal T cell expressed and secreted (RANTES), IL-8, growth-related oncogene α (GRO-α), and I-309) and anti-inflammatory (IL-10) mediators were measured to determine whether the CAR operates appropriately in MDMs upregulated for α7-nAChR. Theoretically, it is to be expected that the higher the levels of α7-nAChR expressed by MDMs, the more accentuated the anti-inflammatory response should be (once activated by ACh). Surprisingly, the addition of ACh did not decrease the production of proinflammatory cytokines (except MCP-1) in MDMs despite elevated levels of α7-nAChR, suggesting a CAR disruption (Figure 1B). Whether the elevated level of α7-nAChR is responsible for disrupting an innate immune mechanism to control inflammation—the CAR—remains to be determined. Interestingly, these results suggest that HIV inactivates this unique innate cellular mechanism in macrophages to ensure elevated levels of interleukins and chemokines, which facilitate HIV pathogenesis and disease progression [133]. Importantly, application of bupropion, an α7-nAChR antagonist, partially rescued the CAR by decreasing some chemokines (RANTES and GRO-α) but not interleukins [107]. Therefore, although important, there are only a limited number of studies aimed at understanding the effects of HIV infection on brain macrophages (microglia), and only a single study has studied its effects on human MDMs. Nevertheless, both peripheral studies identified the α7-nAChR as a key player, which is extremely important because, as described, its activation substantially mitigates inflammation through the CAR.

Also pertinent is the status of the macrophages that directly participate in the CAP: the spleen resident macrophages. In fact, the spleen is not exempt from the systemic damages caused by HIV, and the resident macrophages are irremediably infected (Figure 1B). Studies performed in a macaque SIV model demonstrated that their spleen contains elevated levels of IFN-α together with a higher presence of infected CD4^+^ T lymphocytes and macrophages than their brain and lungs [134], coupled with abundant SIV antigens in their spleen [135]. Similarly, a study performed using an accelerated SIV-infected macaque model reports that, in the spleen, the loss of CD68 and CD163^+^ CD68^+^ macrophages together with an increase in CD163-expressing cells was irreversible, beginning during acute infection and persisting until terminal disease. Interestingly, CD68, CD163, and Mac387 macrophages were highly infected, which primarily occurred in the spleen’s red pulp independent of T lymphocytes. Also, few macrophages underwent apoptosis, indicating that they are a long-lasting target for HIV/SIV [136]. On the other hand, in humans, spleen dendritic cells and CD4^+^ T lymphocytes become infected [137] and trigger inflammatory processes that could explain the follicular hyperplasia and severe cellular depletion of T lymphocyte areas with concomitant plasmacytosis and occurrence of small giant cells [138]. This, in turn, could result in the common splenomegaly detected on the spleen of these individuals [139] and impaired activation of the spleen’s macrophages [140]. Finally, HIV also seems to affect splenic nerve integrity since a cocktail of murine retroviruses, like HIV, destroys splenic sympathetic nerve fibers [141]. Altogether, the damage caused by HIV together with the cellular alterations made to immune cells, mainly macrophages, represents an additional burden that may affect normal functioning of the CAP. Still, it remains uncertain whether the CAP functions normally in productive HIV-infected macrophages.

## 6. Targeting Macrophages to Reduce HIV-Induced Inflammation through α7-nAChRs

Macrophages are important producers of cytokines (interleukins and chemokines) that contribute significantly to the inflammatory phenotype experienced by patients infected with HIV. Therefore, it is prudent to assume that pharmacological strategies targeting the cellular and molecular machinery that governs the macrophage’s secretion of cytokines would be a viable therapeutic alternative. The deactivation (attenuation of inflammatory cytokine production) is an area of great pharmacological interest because it offers the opportunity to modulate the macrophage’s proinflammatory phenotype under infection states. The purpose is to manage the macrophage pharmacologically until reaching an appropriate level of cytokine secretion sufficient to eradicate the pathogen without affecting organs. Of note, the presence of α7-nAChRs in macrophages adds another layer of possible pharmacological intervention through the use of agonists, partial agonists, or positive allosteric modulators (PAMs) (Table 1).

Since its discovery in the CNS, the α7-nAChR has been widely studied and characterized pharmacologically, but not so in macrophages. In fact, there is still controversy whether in macrophages the α7-nAChR behaves as an ion-conducting channel as it does in neurons [142,143]. However, electrophysiological evidence has shown that the α7-nAChR from primary macrophages has ion-channel translocator activity [144], but, apparently, this receptor is hindered by transmembrane/intracellular regulatory proteins that avoid detection of whole-cell patch clamp currents [144]. Thus, the pharmacological properties of this receptor appear to be distinct from those present in the CNS, which is not surprising because, recently, it was shown that α7-nAChR interacts with a number of intracellular proteins and G-protein coupled receptors [145,146,147,148,149] that could modulate or influence its ion channel activity. Moreover, to add an extra level of molecular complexity, macrophages express a partial duplication of α7-nAChR called the dupα7 [150,151] that negatively regulates its activity [151]. Interestingly, studies performed using primary blood-derived human macrophages demonstrate that dupα7 mRNA levels are higher than those of α7-nAChR [151], which is the opposite of what occurs at the protein level. In addition, both IL-1β and LPS are able to reduce dupα7 mRNA levels [151], suggesting that a proinflammatory cytokine and an inflammation inductor could inhibit the expression of dupα7 at the protein level, thereby removing its repressive pressure over α7-nAChR and ultimately facilitating the increase of functional α7-nAChRs available to be activated to alleviate inflammation. In view of these findings, more studies are needed to identify the pool of interacting molecules that influence α7-nAChR activity in macrophages in order to understand and exploit this receptor as a pharmacological target. Also, additional studies focused on the role/effect of dupα7 on inflammation are critical to better understand and design new therapeutic molecules to effectively activate the α7-nAChR in macrophages to control inflammation. Furthermore, several questions regarding dupα7 remain. Is it possible to activate the dupα7 pharmacologically? How does the dupα7 influence pharmacological activation of α7-nAChR in macrophages? Is the dupα7 essential for CAP operation? 

Because of the importance of macrophages as an epicenter of potential pharmacological interventions aimed at the resolution of inflammation, several pharmacological attempts have been made to activate the CAR using in vivo and in vitro approaches (Table 1). Table 1 lists agonists, partial agonists, and PAMs that have been employed in inflammatory settings with macrophage involvement and have shown promising results that warrant their testing in future clinical trials. In fact, some of them are currently under testing [152]. Moreover, several compounds with affinity for the α7-nAChR expressed by macrophages have been tested for different diseases, but they have not yet been considered for testing in macrophages to attenuate inflammation through the activation of CAR. These candidates are agonists (ABT-126, SEN34625/WYE-103914, SEN12333/WAY-317538) partial agonists (RG3487, TC-5619, EVP-6124 (Encenicline), AQW051, BMS-933043), and PAMs (JNJ-39393406, AVL-3288 (UCI-4083) of the α7-nAChR. Therefore, as long as pharmaceutical industries and independent researchers continue to generate new drugs targeting the α7-nAChR, the use of macrophages as a therapeutic target becomes ever more likely.

## 7. Conclusions

Although the discovery of macrophages dates back to the 19th century, these cells continue to surprise the scientific community with their multiplicity of roles in the immune response. Undoubtedly, a critical element of macrophages is their ability to secrete inflammatory cytokines that support and amplify the immune response required to destroy and remove foreign invaders. Importantly, during challenging situations such as HIV infection, macrophages secrete disproportionally high levels of inflammatory cytokines, which then provoke chronic inflammation in these patients. Paradoxically, macrophages also express α7-nAChR, which, upon activation, deactivates the release of proinflammatory cytokines from these cells through the CAR, thereby calibrating the inflammatory response. Nevertheless, it is worth noting that other cellular mechanisms different from those dependent on α7-nAChR can undoubtedly play a fundamental role in relieving inflammation. In fact, intrinsic anti-inflammatory responses in macrophages represent additional therapeutic targets to counteract inflammation. Such is the case of melanocortin (MC) receptors MC_1_ and MC_3_ which modulate macrophage reactivity and reduce inflammation ranging from skin inflammation to reperfusion and joint disease [169]. Moreover, macrophage efferocytosis (phagocytosis of apoptotic cells) is linked to inflammation resolution as demonstrated in post-myocardial infarction and atherosclerosis diseases [170], and can be pharmacologically enhanced by lovastatin [171]. Finally, there are also endogenously produced molecules (lipoxins, resolvins, protectins, and annexins) that can also mediate resolution of inflammation in macrophages [172] that need further examination.

At present, only a limited number of studies have investigated the CAR and the role of macrophages in settings reminiscent of HIV infection. Therefore, a combined approach is essential for exploration of the α7-nAChR in macrophages as a therapeutic target. Fortunately, these efforts can exploit the ongoing development of new agonists, partial agonists, and PAMs targeting α7-nAChR that are presently aimed at treating CNS diseases such as Alzheimer’s disease, Parkinson’s disease, and schizophrenia [152]. These therapeutic candidates should now be tested at the peripheral level to activate α7-nAChR expressed by macrophages for subsequent activation of the CAR. Certainly, research of this type will not be free of challenges, since the activity of α7-nAChR appears to be regulated and influenced by proteins within the cell that interact with this receptor and alter its capacity to respond to stimuli [144,145]. Nevertheless, HIV-infected individuals urgently need novel approaches to counteract chronic inflammation, and α7-nAChR is now emerging as a formidable target that can prevent the development of non-AIDS-related disorders. In fact, stimulation of the vagus nerve, the major neural tract that releases acetylcholine to activate α7-nAChR in macrophages, is being considered as a potential strategy for the treatment of HIV-associated depression [173]. Notably, depression states correlates with inflammation in noninfected [174] and HIV-infected individuals despite antiretroviral therapy [175,176] and may contribute to disease progression [177]. 

New treatment strategies are clearly needed to alleviate HIV-induced inflammation, and macrophages possess a unique target, α7-nAChR, which should be characterized exhaustively in future pharmacologic studies. For example, repurposing drugs such as those presented above may result in new strategies to address inflammation targeting macrophages.

## Figures and Tables

**Table 1:** Drugs that have proved effective in reducing macrophages’ cytokine production through alpha7 nicotinic acetylcholine receptor (α7-nAChR).

Drug	Type of Drug	Effects on Inflammation	Experimental Model/Cell Type/Cell Line	Reference(s)
CNI-1493	Agonist	↓ IL-6	Rat model of vasospasm.	[153]
GTS-21 (DMXB-A)	Agonist	↓ IL-6 ↓ TNF-α ↓ TNF-α and IL-1β ↓ IL-1β ↓ high mobility group box-1 ↓ IL-1β, IL-6, and TNF-α	Mice models of pancreatitis. Alveolar macrophages (in vivo and in vitro). Primary human monocytes and lipopolysaccharide (LPS)-stimulated whole blood obtained from patients with severe sepsis. Human whole blood activated by LPS. Hyperoxic macrophages from a ventilator-associated pneumonia mouse model. LPS-stimulated RAW 264.7 mouse macrophage-like cells.	[132,154,155,156,157,158,159]
AR-R17779	Agonist	↓ TNF-α ↓ TNF-α ↓ IL-1β, IL-6, and MCP-1	Cholinergic anti-inflammatory response (CAR) activation in peritoneal macrophages from postoperative ileus mice models. Brain macrophages (microglia) in neonatal brains from mice. Aorta inflammation in mice.	[160,161,162]
Tropisetron	Partial Agonist	↓ TNF-α and IL-1β	LPS-stimulated primary human monocytes.	[163,164,165,166]
PNU-120596	PAM	↓ TNF-α and IL-6 ↓ TNF-α and IL-6	Rats with hind paw inflammation. Rats modeling ischemia–reperfusion.	[167,168]

↓ = decrease

## References

[1] Mečnikov I.I. (1892). Leçons sur la pathologie comparée de l’inflammation: Faites à l’Institut Pasteur en avril et mai 1891/par Élie Metchnikoff.

[2] Highleyman L. (2010). Inflammation, immune activation, and HIV. BETA Bull. Exp. Treat. AIDS Publ. San Franc. AIDS Found..

[3] Deeks S.G., Tracy R., Douek D.C. (2013). Systemic Effects of Inflammation on Health during Chronic HIV Infection. Immunity.

[4] Wang H., Yu M., Ochani M., Amella C.A., Tanovic M., Susarla S., Li J.H., Wang H., Yang H., Ulloa L. (2003). Nicotinic acetylcholine receptor alpha7 subunit is an essential regulator of inflammation. Nature.

[5] Borovikova L.V., Ivanova S., Zhang M., Yang H., Botchkina G.I., Watkins L.R., Wang H., Abumrad N., Eaton J.W., Tracey K.J. (2000). Vagus nerve stimulation attenuates the systemic inflammatory response to endotoxin. Nature.

[6] Famm K., Litt B., Tracey K.J., Boyden E.S., Slaoui M. (2013). Drug discovery: A jump-start for electroceuticals. Nature.

[7] Torres-Rosas R., Yehia G., Peña G., Mishra P., del Rocio Thompson-Bonilla M., Moreno-Eutimio M.A., Arriaga-Pizano L.A., Isibasi A., Ulloa L. (2014). Dopamine mediates vagal modulation of the immune system by electroacupuncture. Nat. Med..

[8] Ulloa L., Quiroz-Gonzalez S., Torres-Rosas R. (2017). Nerve Stimulation: Immunomodulation and Control of Inflammation. Trends Mol. Med..

[9] Appay V., Sauce D. (2008). Immune activation and inflammation in HIV-1 infection: Causes and consequences. J. Pathol..

[10] Hunt P.W. (2012). HIV and inflammation: Mechanisms and consequences. Curr. HIV/AIDS Rep..

[11] Hileman C.O., Funderburg N.T. (2017). Inflammation, Immune Activation, and Antiretroviral Therapy in HIV. Curr. HIV/AIDS Rep..

[12] Hove-Skovsgaard M., Gaardbo J.C., Kolte L., Winding K., Seljeflot I., Svardal A., Berge R.K., Gerstoft J., Ullum H., Trøseid M., Nielsen S.D. (2017). HIV-infected persons with type 2 diabetes show evidence of endothelial dysfunction and increased inflammation. BMC Infect. Dis..

[13] Van Furth R., Cohn Z.A., Hirsch J.G., Humphrey J.H., Spector W.G., Langevoort H.L. (1972). Mononuclear phagocytic system: New classification of macrophages, monocytes and of their cell line. Bull. World Health Organ..

[14] Yona S., Gordon S. (2015). From the Reticuloendothelial to Mononuclear Phagocyte System—The Unaccounted Years. Front. Immunol..

[15] Ziegler-Heitbrock L., Ancuta P., Crowe S., Dalod M., Grau V., Hart D.N., Leenen P.J.M., Liu Y.-J., MacPherson G., Randolph G.J. (2010). Nomenclature of monocytes and dendritic cells in blood. Blood.

[16] Ziegler-Heitbrock L. (2007). The CD14^+^ CD16^+^ blood monocytes: Their role in infection and inflammation. J. Leukoc. Biol..

[17] Patel A.A., Zhang Y., Fullerton J.N., Boelen L., Rongvaux A., Maini A.A., Bigley V., Flavell R.A., Gilroy D.W., Asquith B. (2017). The fate and lifespan of human monocyte subsets in steady state and systemic inflammation. J. Exp. Med..

[18] Passlick B., Flieger D., Ziegler-Heitbrock H.W. (1989). Identification and characterization of a novel monocyte subpopulation in human peripheral blood. Blood.

[19] Wong K.L., Tai J.J.-Y., Wong W.-C., Han H., Sem X., Yeap W.-H., Kourilsky P., Wong S.-C. (2011). Gene expression profiling reveals the defining features of the classical, intermediate, and nonclassical human monocyte subsets. Blood.

[20] Monie T.P. (2017). Section 1—A Snapshot of the Innate Immune System. The Innate Immune System.

[21] Ginhoux F., Greter M., Leboeuf M., Nandi S., See P., Gokhan S., Mehler M.F., Conway S.J., Ng L.G., Stanley E.R. (2010). Fate mapping analysis reveals that adult microglia derive from primitive macrophages. Science.

[22] Schulz C., Gomez Perdiguero E., Chorro L., Szabo-Rogers H., Cagnard N., Kierdorf K., Prinz M., Wu B., Jacobsen S.E.W., Pollard J.W. (2012). A lineage of myeloid cells independent of Myb and hematopoietic stem cells. Science.

[23] Guilliams M., De Kleer I., Henri S., Post S., Vanhoutte L., De Prijck S., Deswarte K., Malissen B., Hammad H., Lambrecht B.N. (2013). Alveolar macrophages develop from fetal monocytes that differentiate into long-lived cells in the first week of life via GM-CSF. J. Exp. Med..

[24] Yona S., Kim K.-W., Wolf Y., Mildner A., Varol D., Breker M., Strauss-Ayali D., Viukov S., Guilliams M., Misharin A. (2013). Fate mapping reveals origins and dynamics of monocytes and tissue macrophages under homeostasis. Immunity.

[25] Mass E., Ballesteros I., Farlik M., Halbritter F., Günther P., Crozet L., Jacome-Galarza C.E., Händler K., Klughammer J., Kobayashi Y. (2016). Specification of tissue-resident macrophages during organogenesis. Science.

[26] Gordon S. (2016). Phagocytosis: The Legacy of Metchnikoff. Cell.

[27] Ginhoux F., Jung S. (2014). Monocytes and macrophages: Developmental pathways and tissue homeostasis. Nat. Rev. Immunol..

[28] Krenkel O., Tacke F. (2017). Liver macrophages in tissue homeostasis and disease. Nat. Rev. Immunol..

[29] Sieweke M.H., Allen J.E. (2013). Beyond stem cells: Self-renewal of differentiated macrophages. Science.

[30] Geissmann F., Manz M.G., Jung S., Sieweke M.H., Merad M., Ley K. (2010). Development of monocytes, macrophages and dendritic cells. Science.

[31] Arango Duque G., Descoteaux A. (2014). Macrophage Cytokines: Involvement in Immunity and Infectious Diseases. Front. Immunol..

[32] Wynn T.A., Chawla A., Pollard J.W. (2013). Origins and Hallmarks of Macrophages: Development, Homeostasis, and Disease. Nature.

[33] Italiani P., Boraschi D. (2014). From Monocytes to M1/M2 Macrophages: Phenotypical vs. Functional Differentiation. Front. Immunol..

[34] Rőszer T. Understanding the Mysterious M2 Macrophage through Activation Markers and Effector Mechanisms. https://www.hindawi.com/journals/mi/2015/816460/.

[35] Wang Q., Ni H., Lan L., Wei X., Xiang R., Wang Y. (2010). Fra-1 protooncogene regulates IL-6 expression in macrophages and promotes the generation of M2d macrophages. Cell Res..

[36] Ferrante C.J., Pinhal-Enfield G., Elson G., Cronstein B.N., Hasko G., Outram S., Leibovich S.J. (2013). The adenosine-dependent angiogenic switch of macrophages to an M2-like phenotype is independent of interleukin-4 receptor alpha (IL-4Rα) signaling. Inflammation.

[37] Charles A Janeway J., Travers P., Walport M., Shlomchik M.J. (2001). Receptors of the innate immune system. Immunobiology: The Immune System in Health and Disease.

[38] Lim J.J., Grinstein S., Roth Z. (2017). Diversity and Versatility of Phagocytosis: Roles in Innate Immunity, Tissue Remodeling, and Homeostasis. Front. Cell. Infect. Microbiol..

[39] Fact Sheet—Latest Statistics on the Status of the AIDS Epidemic. http://www.unaids.org/en/resources/fact-sheet.

[40] UNAIDS DATA 2017. http://www.unaids.org/en/resources/documents/2017/2017_data_book.

[41] Alkhatib G., Combadiere C., Broder C.C., Feng Y., Kennedy P.E., Murphy P.M., Berger E.A. (1996). CC CKR5: A RANTES, MIP-1alpha, MIP-1beta receptor as a fusion cofactor for macrophage-tropic HIV-1. Science.

[42] Choe H., Farzan M., Sun Y., Sullivan N., Rollins B., Ponath P.D., Wu L., Mackay C.R., LaRosa G., Newman W. (1996). The beta-chemokine receptors CCR3 and CCR5 facilitate infection by primary HIV-1 isolates. Cell.

[43] Cocchi F., DeVico A.L., Garzino-Demo A., Arya S.K., Gallo R.C., Lusso P. (1995). Identification of RANTES, MIP-1 alpha, and MIP-1 beta as the major HIV-suppressive factors produced by CD8^+^ T cells. Science.

[44] Deng H., Liu R., Ellmeier W., Choe S., Unutmaz D., Burkhart M., Di Marzio P., Marmon S., Sutton R.E., Hill C.M. (1996). Identification of a major co-receptor for primary isolates of HIV-1. Nature.

[45] Feng Y., Broder C.C., Kennedy P.E., Berger E.A. (1996). HIV-1 entry cofactor: Functional cDNA cloning of a seven-transmembrane, G protein-coupled receptor. Science.

[46] Schmidtmayerova H., Sherry B., Bukrinsky M. (1996). Chemokines and HIV replication. Nature.

[47] Langford S.E., Ananworanich J., Cooper D.A. (2007). Predictors of disease progression in HIV infection: A review. AIDS Res. Ther..

[48] Ganesan A., Chattopadhyay P.K., Brodie T.M., Qin J., Gu W., Mascola J.R., Michael N.L., Follmann D.A., Roederer M. (2010). Immunological and Virological Events in Early HIV Infection Predict Subsequent Rate of Progression. J. Infect. Dis..

[49] Lackner A.A., Mohan M., Veazey R.S. (2009). The Gastrointestinal Tract and AIDS Pathogenesis. Gastroenterology.

[50] Brenchley J.M., Douek D.C. (2008). HIV infection and the gastrointestinal immune system. Mucosal Immunol..

[51] Deeks S.G., Kitchen C.M.R., Liu L., Guo H., Gascon R., Narváez A.B., Hunt P., Martin J.N., Kahn J.O., Levy J. (2004). Immune activation set point during early HIV infection predicts subsequent CD4^+^ T-cell changes independent of viral load. Blood.

[52] Hazenberg M.D., Otto S.A., van Benthem B.H.B., Roos M.T.L., Coutinho R.A., Lange J.M.A., Hamann D., Prins M., Miedema F. (2003). Persistent immune activation in HIV-1 infection is associated with progression to AIDS. AIDS Lond. Engl..

[53] Brenchley J.M., Price D.A., Schacker T.W., Asher T.E., Silvestri G., Rao S., Kazzaz Z., Bornstein E., Lambotte O., Altmann D. (2006). Microbial translocation is a cause of systemic immune activation in chronic HIV infection. Nat. Med..

[54] Dillon S.M., Frank D.N., Wilson C.C. (2016). The gut microbiome and HIV-1 pathogenesis: A two-way street. AIDS Lond. Engl..

[55] Dubourg G., Surenaud M., Lévy Y., Hüe S., Raoult D. (2017). Microbiome of HIV-infected people. Microb. Pathog..

[56] Zilberman-Schapira G., Zmora N., Itav S., Bashiardes S., Elinav H., Elinav E. (2016). The gut microbiome in human immunodeficiency virus infection. BMC Med..

[57] Douek D. (2007). HIV disease progression: Immune activation, microbes, and a leaky gut. Top. HIV Med. Publ. Int. AIDS Soc. USA.

[58] Wallet M.A., Rodriguez C.A., Yin L., Saporta S., Chinratanapisit S., Hou W., Sleasman J.W., Goodenow M.M. (2010). Microbial translocation induces persistent macrophage activation unrelated to HIV-1 levels or T-cell activation following therapy. AIDS Lond. Engl..

[59] Kelesidis T., Kendall M.A., Yang O.O., Hodis H.N., Currier J.S. (2012). Biomarkers of Microbial Translocation and Macrophage Activation: Association With Progression of Subclinical Atherosclerosis in HIV-1 Infection. J. Infect. Dis..

[60] Stacey A.R., Norris P.J., Qin L., Haygreen E.A., Taylor E., Heitman J., Lebedeva M., DeCamp A., Li D., Grove D. (2009). Induction of a striking systemic cytokine cascade prior to peak viremia in acute human immunodeficiency virus type 1 infection, in contrast to more modest and delayed responses in acute hepatitis B and C virus infections. J. Virol..

[61] Roberts L., Passmore J.-A.S., Williamson C., Little F., Bebell L.M., Mlisana K., Burgers W.A., van Loggerenberg F., Walzl G., Djoba Siawaya J.F. (2010). Plasma cytokine levels during acute HIV-1 infection predict HIV disease progression. AIDS Lond. Engl..

[62] Mugwe J.N., Gicheru M.M., Mwatha J. (2016). Plasma Cytokine Profiles as Predictive Biomarkers of HIV and Aids Progression among HIV Patients Attending Nakuru Provincial General Hospital, Kenya. Am. J. Med. Biol. Res. Am. J. Med. Biol. Res..

[63] Shebl F.M., Yu K., Landgren O., Goedert J.J., Rabkin C.S. (2012). Increased Levels of Circulating Cytokines with HIV-Related Immunosuppression. AIDS Res. Hum. Retroviruses.

[64] Tien P.C., Choi A.I., Zolopa A.R., Benson C., Tracy R., Scherzer R., Bacchetti P., Shlipak M., Grunfeld C. (2010). Inflammation and mortality in HIV-infected adults: Analysis of the FRAM study cohort. J. Acquir. Immune Defic. Syndr. 1999.

[65] Baker J.V., Neuhaus J., Duprez D., Kuller L.H., Tracy R., Belloso W.H., De Wit S., Drummond F., Lane H.C., Ledergerber B. (2011). Changes in inflammatory and coagulation biomarkers: A randomized comparison of immediate versus deferred antiretroviral therapy in patients with HIV infection. J. Acquir. Immune Defic. Syndr. 1999.

[66] McDonald B., Moyo S., Gabaitiri L., Gaseitsiwe S., Bussmann H., Koethe J.R., Musonda R., Makhema J., Novitsky V., Marlink R.G. (2013). Persistently elevated serum interleukin-6 predicts mortality among adults receiving combination antiretroviral therapy in Botswana: Results from a clinical trial. AIDS Res. Hum. Retroviruses.

[67] Deeks S.G. (2011). HIV infection, inflammation, immunosenescence, and aging. Annu. Rev. Med..

[68] Caruana G., Vidili G., Serra P.A., Bagella P., Spanu A., Fiore V., Calvisi D.F., Manetti R., Rocchitta G., Nuvoli S. (2017). The burden of HIV-associated neurocognitive disorder (HAND) in post-HAART era: A multidisciplinary review of the literature. Eur. Rev. Med. Pharmacol. Sci..

[69] Phair J., Palella F. (2011). Renal disease in HIV infected Individuals. Curr. Opin. HIV AIDS.

[70] Compston J. (2016). HIV infection and bone disease. J. Intern. Med..

[71] Farahani M., Mulinder H., Farahani A., Marlink R. (2017). Prevalence and distribution of non-AIDS causes of death among HIV-infected individuals receiving antiretroviral therapy: A systematic review and meta-analysis. Int. J. STD AIDS.

[72] Wong C., Gange S.J., Moore R.D., Justice A.C., Buchacz K., Abraham A.G., Rebeiro P.F., Koethe J.R., Martin J.N., Horberg M.A. (2017). Multimorbidity among Persons Living with HIV in the U.S. Clin. Infect. Dis. Off. Publ. Infect. Dis. Soc. Am..

[73] Ruan A., Tobin N.H., Mulligan K., Rollie A., Li F., Sleasman J., Aldrovandi G.M. (2016). Brief Report: Macrophage Activation in HIV-Infected Adolescent Males Contributes to Differential Bone Loss by Sex Adolescent Trials Network Study 021. JAIDS J. Acquir. Immune Defic. Syndr..

[74] Kearns A., Gordon J., Burdo T.H., Qin X. (2017). HIV-1-Associated Atherosclerosis: Unraveling the Missing Link. J. Am. Coll. Cardiol..

[75] Hanna D.B., Lin J., Post W.S., Hodis H.N., Xue X., Anastos K., Cohen M.H., Gange S.J., Haberlen S.A., Heath S.L. (2017). Association of Macrophage Inflammation Biomarkers With Progression of Subclinical Carotid Artery Atherosclerosis in HIV-Infected Women and Men. J. Infect. Dis..

[76] Shaked I., Hanna D.B., Gleißner C., Marsh B., Plants J., Tracy D., Anastos K., Cohen M., Golub E.T., Karim R. (2014). Macrophage inflammatory markers are associated with subclinical carotid artery disease in women with human immunodeficiency virus or hepatitis C virus infection. Arterioscler. Thromb. Vasc. Biol..

[77] Ellis R.J., Badiee J., Vaida F., Letendre S., Heaton R.K., Clifford D., Collier A.C., Gelman B., McArthur J., Morgello S. (2011). CD4 nadir is a predictor of HIV neurocognitive impairment in the era of combination antiretroviral therapy. AIDS Lond. Engl..

[78] Fischer-Smith T., Croul S., Sverstiuk A.E., Capini C., L’Heureux D., Régulier E.G., Richardson M.W., Amini S., Morgello S., Khalili K., Rappaport J. (2001). CNS invasion by CD14+/CD16+ peripheral blood-derived monocytes in HIV dementia: Perivascular accumulation and reservoir of HIV infection. J. Neurovirol..

[79] Lyons J.L., Uno H., Ancuta P., Kamat A., Moore D.J., Singer E.J., Morgello S., Gabuzda D. (2011). Plasma sCD14 is a biomarker associated with impaired neurocognitive test performance in attention and learning domains in HIV infection. J. Acquir. Immune Defic. Syndr. 1999.

[80] McCombe J.A., Vivithanaporn P., Gill M.J., Power C. (2013). Predictors of symptomatic HIV-associated neurocognitive disorders in universal health care. HIV Med..

[81] Rappaport J., Volsky D.J. (2015). Role of the Macrophage in HIV-Associated Neurocognitive Disorders and Other Comorbidities in Patients on Effective Antiretroviral Treatment. J. Neurovirol..

[82] Hassan J., Browne K., De Gascun C. (2016). HIV-1 in Monocytes and Macrophages: An Overlooked Reservoir?. Viral Immunol..

[83] Cribbs S.K., Lennox J., Caliendo A.M., Brown L.A., Guidot D.M. (2015). Healthy HIV-1-infected individuals on highly active antiretroviral therapy harbor HIV-1 in their alveolar macrophages. AIDS Res. Hum. Retroviruses.

[84] Ray P.E., Liu X.H., Henry D., Dye L., Xu L., Orenstein J.M., Schuztbank T.E. (1998). Infection of human primary renal epithelial cells with HIV-1 from children with HIV-associated nephropathy. Kidney Int..

[85] O’Donnell M.P., Chao C.C., Gekker G., Modi K.S., Kasiske B.L., Keane W.F. (1998). Renal cell cytokine production stimulates HIV-1 expression in chronically HIV-1-infected monocytes. Kidney Int..

[86] Reid M., Ma Y., Scherzer R., Price J.C., French A.L., Plankey M.W., Grunfeld C., Tien P.C. (2017). Higher CD163 levels are associated with insulin resistance in hepatitis C virus-infected and HIV-infected adults. AIDS Lond. Engl..

[87] (2010). Macrophages linked to lymphoma. AIDS Patient Care STDs.

[88] Huysentruyt L.C., McGrath M.S. (2010). The role of macrophages in the development and progression of AIDS-related non-Hodgkin lymphoma. J. Leukoc. Biol..

[89] Hosoi T., Okuma Y., Matsuda T., Nomura Y. (2005). Novel pathway for LPS-induced afferent vagus nerve activation: Possible role of nodose ganglion. Auton. Neurosci. Basic Clin..

[90] Ek M., Kurosawa M., Lundeberg T., Ericsson A. (1998). Activation of vagal afferents after intravenous injection of interleukin-1beta: Role of endogenous prostaglandins. J. Neurosci..

[91] Van der Kleij H., Charles N., Karimi K., Mao Y.-K., Foster J., Janssen L., Yang P.C., Kunze W., Rivera J., Bienenstock J. (2010). Evidence for neuronal expression of functional Fc (ε and γ) receptors. J. Allergy Clin. Immunol..

[92] Page A.J., O’Donnell T.A., Blackshaw L.A. (2000). P2X purinoceptor-induced sensitization of ferret vagal mechanoreceptors in oesophageal inflammation. J. Physiol..

[93] Niijima A. (1996). The afferent discharges from sensors for interleukin 1 beta in the hepatoportal system in the anesthetized rat. J. Auton. Nerv. Syst..

[94] Inoue T., Tanaka S., Okusa M.D. (2017). Neuroimmune Interactions in Inflammation and Acute Kidney Injury. Front. Immunol..

[95] Lang P.M., Tracey D.J., Irnich D., Sippel W., Grafe P. (2002). Activation of adenosine and P2Y receptors by ATP in human peripheral nerve. Naunyn Schmiedebergs Arch. Pharmacol..

[96] Pavlov V.A., Ochani M., Gallowitsch-Puerta M., Ochani K., Huston J.M., Czura C.J., Al-Abed Y., Tracey K.J. (2006). Central muscarinic cholinergic regulation of the systemic inflammatory response during endotoxemia. Proc. Natl. Acad. Sci. USA.

[97] Pavlov V.A., Parrish W.R., Rosas-Ballina M., Ochani M., Puerta M., Ochani K., Chavan S., Al-Abed Y., Tracey K.J. (2009). Brain acetylcholinesterase activity controls systemic cytokine levels through the cholinergic anti-inflammatory pathway. Brain Behav. Immun..

[98] Langley R.J., Kalra R., Mishra N.C., Sopori M.L. (2004). Central but not the peripheral action of cholinergic compounds suppresses the immune system. J. Neuroimmunol..

[99] Rosas-Ballina M., Olofsson P.S., Ochani M., Valdés-Ferrer S.I., Levine Y.A., Reardon C., Tusche M.W., Pavlov V.A., Andersson U., Chavan S. (2011). Acetylcholine-synthesizing T cells relay neural signals in a vagus nerve circuit. Science.

[100] Chernyavsky A.I., Arredondo J., Skok M., Grando S.A. (2010). Auto/paracrine control of inflammatory cytokines by acetylcholine in macrophage-like U937 cells through nicotinic receptors. Int. Immunopharmacol..

[101] Galvis G., Lips K.S., Kummer W. (2006). Expression of nicotinic acetylcholine receptors on murine alveolar macrophages. J. Mol. Neurosci..

[102] Kim S.-S., Ye C., Kumar P., Chiu I., Subramanya S., Wu H., Shankar P., Manjunath N. (2010). Targeted Delivery of siRNA to Macrophages for Anti-inflammatory Treatment. Mol. Ther..

[103] Shytle R.D., Mori T., Townsend K., Vendrame M., Sun N., Zeng J., Ehrhart J., Silver A.A., Sanberg P.R., Tan J. (2004). Cholinergic modulation of microglial activation by alpha 7 nicotinic receptors. J. Neurochem..

[104] Mikulski Z., Hartmann P., Jositsch G., Zasłona Z., Lips K.S., Pfeil U., Kurzen H., Lohmeyer J., Clauss W.G., Grau V. (2010). Nicotinic receptors on rat alveolar macrophages dampen ATP-induced increase in cytosolic calcium concentration. Respir. Res..

[105] Van der Zanden E.P., Snoek S.A., Heinsbroek S.E., Stanisor O.I., Verseijden C., Boeckxstaens G.E., Peppelenbosch M.P., Greaves D.R., Gordon S., De Jonge W.J. (2009). Vagus nerve activity augments intestinal macrophage phagocytosis via nicotinic acetylcholine receptor α4β2. Gastroenterology.

[106] Nemethova A., Michel K., Gomez-Pinilla P.J., Boeckxstaens G.E., Schemann M. (2013). Nicotine Attenuates Activation of Tissue Resident Macrophages in the Mouse Stomach through the β2 Nicotinic Acetylcholine Receptor. PLoS ONE.

[107] Delgado-Vélez M., Báez-Pagán C.A., Gerena Y., Quesada O., Santiago-Pérez L.I., Capó-Vélez C.M., Wojna V., Meléndez L., León-Rivera R., Silva W., Lasalde-Dominicci J.A. (2015). The α7-nicotinic receptor is upregulated in immune cells from HIV-seropositive women: Consequences to the cholinergic anti-inflammatory response. Clin. Transl. Immunol..

[108] Paiardini M., Müller-Trutwin M. (2013). HIV-associated chronic immune activation. Immunol. Rev..

[109] Li Q., Estes J.D., Schlievert P.M., Duan L., Brosnahan A.J., Southern P.J., Reilly C.S., Peterson M.L., Schultz-Darken N., Brunner K.G. (2009). Glycerol monolaurate prevents mucosal SIV transmission. Nature.

[110] Manches O., Frleta D., Bhardwaj N. (2014). Dendritic cells in progression and pathology of HIV infection. Trends Immunol..

[111] Funderburg N.T. (2014). Markers of coagulation and inflammation often remain elevated in ART-treated HIV-infected patients. Curr. Opin. HIV AIDS.

[112] Neri P., Bracci L., Rustici M., Santucci A. (1990). Sequence homology between HIV gp120, rabies virus glycoprotein, and snake venom neurotoxins. Is the nicotinic acetylcholine receptor an HIV receptor?. Arch. Virol..

[113] Sönnerborg A., Johansson B. (1993). The neurotoxin-like sequence of human immunodeficiency virus gp120: A comparison of sequence data from patients with and without neurological symptoms. Virus Genes.

[114] Bracci L., Lozzi L., Rustici M., Neri P. (1992). Binding of HIV-1 gp120 to the nicotinic receptor. FEBS Lett..

[115] Bracci L., Ballas S.K., Spreafico A., Neri P. (1997). Molecular mimicry between the rabies virus glycoprotein and human immunodeficiency virus-1 GP120: Cross-reacting antibodies induced by rabies vaccination. Blood.

[116] Yoshikawa H., Kurokawa M., Ozaki N., Nara K., Atou K., Takada E., Kamochi H., Suzuki N. (2006). Nicotine inhibits the production of proinflammatory mediators in human monocytes by suppression of I-kappaB phosphorylation and nuclear factor-kappaB transcriptional activity through nicotinic acetylcholine receptor alpha7. Clin. Exp. Immunol..

[117] Ciardo A., Meldolesi J. (1993). Effects of the HIV-1 envelope glycoprotein gp120 in cerebellar cultures. [Ca^2+^]_i_ increases in a glial cell subpopulation. Eur. J. Neurosci..

[118] Hesselgesser J., Taub D., Baskar P., Greenberg M., Hoxie J., Kolson D.L., Horuk R. (1998). Neuronal apoptosis induced by HIV-1 gp120 and the chemokine SDF-1α is mediated by the chemokine receptor CXCR4. Curr. Biol. CB.

[119] Feligioni M., Raiteri L., Pattarini R., Grilli M., Bruzzone S., Cavazzani P., Raiteri M., Pittaluga A. (2003). The human immunodeficiency virus-1 protein Tat and its discrete fragments evoke selective release of acetylcholine from human and rat cerebrocortical terminals through species-specific mechanisms. J. Neurosci. Off. J. Soc. Neurosci..

[120] Ballester L.Y., Capó-Vélez C.M., García-Beltrán W.F., Ramos F.M., Vázquez-Rosa E., Ríos R., Mercado J.R., Meléndez R.I., Lasalde-Dominicci J.A. (2012). Up-regulation of the neuronal nicotinic receptor α7 by HIV glycoprotein 120: Potential implications for HIV-associated neurocognitive disorder. J. Biol. Chem..

[121] Cao J., Wang S., Wang J., Cui W., Nesil T., Vigorito M., Chang S.L., Li M.D. (2013). RNA deep sequencing analysis reveals that nicotine restores impaired gene expression by viral proteins in the brains of HIV-1 transgenic rats. PLoS ONE.

[122] Zhang B., Yu J.-Y., Liu L.-Q., Peng L., Chi F., Wu C.-H., Jong A., Wang S.-F., Cao H., Huang S.-H. (2015). Alpha7 nicotinic acetylcholine receptor is required for blood-brain barrier injury-related CNS disorders caused by Cryptococcus neoformans and HIV-1 associated comorbidity factors. BMC Infect. Dis..

[123] Ramos F.M., Delgado-Vélez M., Ortiz Á.L., Báez-Pagán C.A., Quesada O., Lasalde-Dominicci J.A. (2015). Expression of CHRFAM7A and CHRNA7 in neuronal cells and postmortem brain of HIV-infected patients: Considerations for HIV-associated neurocognitive disorder. J. Neurovirol..

[124] Liu L., Yu J., Li L., Zhang B., Liu L., Wu C.-H., Jong A., Mao D.-A., Huang S.-H. (2017). Alpha7 nicotinic acetylcholine receptor is required for amyloid pathology in brain endothelial cells induced by Glycoprotein 120, methamphetamine and nicotine. Sci. Rep..

[125] Ekins S., Mathews P., Saito E.K., Diaz N., Naylor D., Chung J., McMurtray A.M. (2017). α7-Nicotinic acetylcholine receptor inhibition by indinavir: Implications for cognitive dysfunction in treated HIV disease. AIDS Lond. Engl..

[126] Capó-Vélez C.M., Morales-Vargas B., García-González A., Grajales-Reyes J.G., Delgado-Vélez M., Madera B., Báez-Pagán C.A., Quesada O., Lasalde-Dominicci J.A. (2018). The alpha7-nicotinic receptor contributes to gp120-induced neurotoxicity: Implications in HIV-associated neurocognitive disorders. Sci. Rep..

[127] Gundavarapu S., Mishra N.C., Singh S.P., Langley R.J., Saeed A.I., Feghali-Bostwick C.A., McIntosh J.M., Hutt J., Hegde R., Buch S. (2013). HIV gp120 induces mucus formation in human bronchial epithelial cells through CXCR4/α7-nicotinic acetylcholine receptors. PLoS ONE.

[128] Catani M.V., Corasaniti M.T., Navarra M., Nisticò G., Finazzi-Agrò A., Melino G. (2000). gp120 induces cell death in human neuroblastoma cells through the CXCR4 and CCR5 chemokine receptors. J. Neurochem..

[129] Giunta B., Ehrhart J., Townsend K., Sun N., Vendrame M., Shytle D., Tan J., Fernandez F. (2004). Galantamine and nicotine have a synergistic effect on inhibition of microglial activation induced by HIV-1 gp120. Brain Res. Bull..

[130] Rock R.B., Gekker G., Aravalli R.N., Hu S., Sheng W.S., Peterson P.K. (2008). Potentiation of HIV-1 expression in microglial cells by nicotine: Involvement of transforming growth factor-beta 1. J. Neuroimmune Pharmacol. Off. J. Soc. NeuroImmune Pharmacol..

[131] Abbud R.A., Finegan C.K., Guay L.A., Rich E.A. (1995). Enhanced production of human immunodeficiency virus type 1 by in vitro-infected alveolar macrophages from otherwise healthy cigarette smokers. J. Infect. Dis..

[132] Loram L.C., Harrison J.A., Chao L., Taylor F.R., Reddy A., Travis C.L., Giffard R., Al-Abed Y., Tracey K., Maier S.F. (2010). Intrathecal injection of an alpha seven nicotinic acetylcholine receptor agonist attenuates gp120-induced mechanical allodynia and spinal pro-inflammatory cytokine profiles in rats. Brain Behav. Immun..

[133] Alfano M., Crotti A., Vicenzi E., Poli G. (2008). New players in cytokine control of HIV infection. Curr. HIV/AIDS Rep..

[134] Zaritsky L.A., Dery A., Leong W.Y., Gama L., Clements J.E. (2013). Tissue-specific interferon alpha subtype response to SIV infection in brain, spleen, and lung. J. Interferon Cytokine Res. Off. J. Int. Soc. Interferon Cytokine Res..

[135] Ward J.M., O’Leary T.J., Baskin G.B., Benveniste R., Harris C.A., Nara P.L., Rhodes R.H. (1987). Immunohistochemical localization of human and simian immunodeficiency viral antigens in fixed tissue sections. Am. J. Pathol..

[136] Williams D.W., Engle E.L., Shirk E.N., Queen S.E., Gama L., Mankowski J.L., Zink M.C., Clements J.E. (2016). Splenic Damage during SIV Infection. Am. J. Pathol..

[137] McIlroy D., Autran B., Cheynier R., Wain-Hobson S., Clauvel J.P., Oksenhendler E., Debré P., Hosmalin A. (1995). Infection frequency of dendritic cells and CD4^+^ T lymphocytes in spleens of human immunodeficiency virus-positive patients. J. Virol..

[138] Falk S., Müller H., Stutte H.J. (1988). The spleen in acquired immunodeficiency syndrome (AIDS). Pathol. Res. Pract..

[139] Ansovini R., Barbolini G., Migaldi M., Botticelli L., De Rienzo B. (1998). AIDS splenomegaly and related iron problems. Pathologica.

[140] Falk S., Stutte H.J. (1990). The spleen in HIV infection—Morphological evidence of HIV-associated macrophage dysfunction. Res. Virol..

[141] Kelley S.P., Moynihan J.A., Stevens S.Y., Grota L.J., Felten D.L. (2003). Sympathetic nerve destruction in spleen in murine AIDS. Brain Behav. Immun..

[142] Skok M.V. (2009). Editorial: To channel or not to channel? Functioning of nicotinic acetylcholine receptors in leukocytes. J. Leukoc. Biol..

[143] Villiger Y., Szanto I., Jaconi S., Blanchet C., Buisson B., Krause K.-H., Bertrand D., Romand J.-A. (2002). Expression of an alpha7 duplicate nicotinic acetylcholine receptor-related protein in human leukocytes. J. Neuroimmunol..

[144] Báez-Pagán C.A., Delgado-Vélez M., Lasalde-Dominicci J.A. (2015). Activation of the Macrophage α7 Nicotinic Acetylcholine Receptor and Control of Inflammation. J. Neuroimmune Pharmacol. Off. J. Soc. NeuroImmune Pharmacol..

[145] Paulo J.A., Brucker W.J., Hawrot E. (2009). Proteomic analysis of an alpha7 nicotinic acetylcholine receptor interactome. J. Proteome Res..

[146] Nordman J.C., Kabbani N. (2012). An interaction between α7 nicotinic receptors and a G-protein pathway complex regulates neurite growth in neural cells. J. Cell Sci..

[147] King J.R., Nordman J.C., Bridges S.P., Lin M.-K., Kabbani N. (2015). Identification and Characterization of a G Protein-binding Cluster in α7 Nicotinic Acetylcholine Receptors. J. Biol. Chem..

[148] King J.R., Kabbani N. (2016). Alpha 7 nicotinic receptor coupling to heterotrimeric G proteins modulates RhoA activation, cytoskeletal motility, and structural growth. J. Neurochem..

[149] King J.R., Gillevet T.C., Kabbani N. (2017). A G protein-coupled α7 nicotinic receptor regulates signaling and TNF-α release in microglia. FEBS Open Bio.

[150] Benfante R., Antonini R.A., De Pizzol M., Gotti C., Clementi F., Locati M., Fornasari D. (2011). Expression of the α7 nAChR subunit duplicate form (CHRFAM7A) is down-regulated in the monocytic cell line THP-1 on treatment with LPS. J. Neuroimmunol..

[151] De Lucas-Cerrillo A.M., Maldifassi M.C., Arnalich F., Renart J., Atienza G., Serantes R., Cruces J., Sánchez-Pacheco A., Andrés-Mateos E., Montiel C. (2011). Function of partially duplicated human α77 nicotinic receptor subunit CHRFAM7A gene: Potential implications for the cholinergic anti-inflammatory response. J. Biol. Chem..

[152] Yang T., Xiao T., Sun Q., Wang K. (2017). The current agonists and positive allosteric modulators of α7 nAChR for CNS indications in clinical trials. Acta Pharm. Sin. B.

[153] Bowman G., Bonneau R.H., Chinchilli V.M., Tracey K.J., Cockroft K.M. (2006). A novel inhibitor of inflammatory cytokine production (CNI-1493) reduces rodent post-hemorrhagic vasospasm. Neurocrit. Care.

[154] van Westerloo D.J., Giebelen I.A., Florquin S., Bruno M.J., Larosa G.J., Ulloa L., Tracey K.J., van der Poll T. (2006). The vagus nerve and nicotinic receptors modulate experimental pancreatitis severity in mice. Gastroenterology.

[155] Giebelen I.A.J., van Westerloo D.J., LaRosa G.J., de Vos A.F., van der Poll T. (2007). Stimulation of alpha 7 cholinergic receptors inhibits lipopolysaccharide-induced neutrophil recruitment by a tumor necrosis factor alpha-independent mechanism. Shock Augusta Ga.

[156] Giebelen I.A.J., van Westerloo D.J., LaRosa G.J., de Vos A.F., van der Poll T. (2007). Local stimulation of alpha7 cholinergic receptors inhibits LPS-induced TNF-alpha release in the mouse lung. Shock Augusta Ga.

[157] Rosas-Ballina M., Goldstein R.S., Gallowitsch-Puerta M., Yang L., Valdés-Ferrer S.I., Patel N.B., Chavan S., Al-Abed Y., Yang H., Tracey K.J. (2009). The Selective α7 Agonist GTS-21 Attenuates Cytokine Production in Human Whole Blood and Human Monocytes Activated by Ligands for TLR2, TLR3, TLR4, TLR9, and RAGE. Mol. Med..

[158] Sitapara R.A., Antoine D.J., Sharma L., Patel V.S., Ashby C.R., Gorasiya S., Yang H., Zur M., Mantell L.L. (2014). The α7 nicotinic acetylcholine receptor agonist GTS-21 improves bacterial clearance in mice by restoring hyperoxia-compromised macrophage function. Mol. Med. Camb. Mass..

[159] Yue Y., Liu R., Cheng W., Hu Y., Li J., Pan X., Peng J., Zhang P. (2015). GTS-21 attenuates lipopolysaccharide-induced inflammatory cytokine production in vitro by modulating the Akt and NF-κB signaling pathway through the α7 nicotinic acetylcholine receptor. Int. Immunopharmacol..

[160] Van Maanen M.A., Lebre M.C., van der Poll T., LaRosa G.J., Elbaum D., Vervoordeldonk M.J., Tak P.P. (2009). Stimulation of nicotinic acetylcholine receptors attenuates collagen-induced arthritis in mice. Arthritis Rheum..

[161] Hua S., Ek C.J., Mallard C., Johansson M.E. (2014). Perinatal hypoxia-ischemia reduces α 7 nicotinic receptor expression and selective α 7 nicotinic receptor stimulation suppresses inflammation and promotes microglial Mox phenotype. BioMed Res. Int..

[162] Watanabe A., Ichiki T., Kojima H., Takahara Y., Hurt-Camejo E., Michaëlsson E., Sankoda C., Ikeda J., Inoue E., Tokunou T. (2016). Suppression of abdominal aortic aneurysm formation by AR-R17779, an agonist for the α7 nicotinic acetylcholine receptor. Atherosclerosis.

[163] Hashimoto K. (2015). Targeting of α7 Nicotinic Acetylcholine Receptors in the Treatment of Schizophrenia and the Use of Auditory Sensory Gating as a Translational Biomarker. Curr. Pharm. Des..

[164] Maehara T., Matsumoto K., Horiguchi K., Kondo M., Iino S., Horie S., Murata T., Tsubone H., Shimada S., Ozaki H. (2015). Therapeutic action of 5-HT3 receptor antagonists targeting peritoneal macrophages in post-operative ileus. Br. J. Pharmacol..

[165] Tasaka Y., Yasunaga D., Kiyoi T., Tanaka M., Tanaka A., Suemaru K., Araki H. (2015). Involvement of stimulation of α7 nicotinic acetylcholine receptors in the suppressive effect of tropisetron on dextran sulfate sodium-induced colitis in mice. J. Pharmacol. Sci..

[166] Fiebich B.L., Akundi R.S., Lieb K., Candelario-Jalil E., Gmeiner D., Haus U., Müller W., Stratz T., Muñoz E. (2004). Antiinflammatory effects of 5-HT3 receptor antagonists in lipopolysaccharide-stimulated primary human monocytes. Scand. J. Rheumatol..

[167] Munro G., Hansen R., Erichsen H., Timmermann D., Christensen J., Hansen H. (2012). The α7 nicotinic ACh receptor agonist compound B and positive allosteric modulator PNU-120596 both alleviate inflammatory hyperalgesia and cytokine release in the rat. Br. J. Pharmacol..

[168] Li H., Zhang Z.-Z., Zhan J., He X.-H., Song X.-M., Wang Y.-L. (2012). Protective effect of PNU-120596, a selective alpha7 nicotinic acetylcholine receptor-positive allosteric modulator, on myocardial ischemia-reperfusion injury in rats. J. Cardiovasc. Pharmacol..

[169] Patel H.B., Montero-Melendez T., Greco K.V., Perretti M. (2011). Melanocortin Receptors as Novel Effectors of Macrophage Responses in Inflammation. Front. Immunol..

[170] Thorp E.B. (2012). Contrasting Inflammation Resolution during Atherosclerosis and Post Myocardial Infarction at the Level of Monocyte/Macrophage Phagocytic Clearance. Front. Immunol..

[171] Morimoto K., Janssen W.J., Fessler M.B., McPhillips K.A., Borges V.M., Bowler R.P., Xiao Y.-Q., Kench J.A., Henson P.M., Vandivier R.W. (2006). Lovastatin enhances clearance of apoptotic cells (efferocytosis) with implications for chronic obstructive pulmonary disease. J. Immunol. Baltim. Md 1950.

[172] Kennedy A., Fearon U., Veale D.J., Godson C. (2011). Macrophages in Synovial Inflammation. Front. Immunol..

[173] Nicholson W.C., Kempf M.-C., Moneyham L., Vance D.E. (2017). The potential role of vagus-nerve stimulation in the treatment of HIV-associated depression: A review of literature. Neuropsychiatr. Dis. Treat..

[174] Skaper S.D., Facci L., Zusso M., Giusti P. (2018). An Inflammation-Centric View of Neurological Disease: Beyond the Neuron. Front. Cell. Neurosci..

[175] Rivera-Rivera Y., García Y., Toro V., Cappas N., López P., Yamamura Y., Rivera-Amill V. (2014). Depression Correlates with Increased Plasma Levels of Inflammatory Cytokines and a Dysregulated Oxidant/Antioxidant Balance in HIV-1-Infected Subjects Undergoing Antiretroviral Therapy. J. Clin. Cell. Immunol..

[176] Norcini Pala A., Steca P., Bagrodia R., Helpman L., Colangeli V., Viale P., Wainberg M.L. (2016). Subtypes of depressive symptoms and inflammatory biomarkers: An exploratory study on a sample of HIV-positive patients. Brain. Behav. Immun..

[177] Rivera-Rivera Y., Vázquez-Santiago F.J., Albino E., Sánchez M.D.C., Rivera-Amill V. (2016). Impact of Depression and Inflammation on the Progression of HIV Disease. J. Clin. Cell. Immunol..

